# The British Journals, &c.: Medical Statistics

**Published:** 1842-04

**Authors:** 


### MEDICAL STATISTICS.

Health of the Labouring Population of Kelso, in two Decennial Periods. This is a valuable paper, which we are induced to notice, in order to furnish practitioners throughout the country with an opportunity of comparing the facts of the author's paper, with those of a like kind which may exist elsewhere. The total number of cases entered for the first decennial period, extending from 1777 to 1787, is 4705; for the second decennium, extending from 1829 to 1839, 8034; making in all 12,739. The total number of deaths is 423.

In a table designed to show the mean relative prevalence of various epidemic and endemic diseases, such as ague, fever, smallpox, chickenpox, scarlet fever, measles, hooping-cough, and erysipelas, and their proportion per cent. to all other diseases, we find that the total amount of these diseases was, in the first decennium, 33·07; in the second, 15·35. Cases of ague appear to bear an extraordinary proportion, in the first decennium, presenting an average of nearly one seventh of all cases coming under treatment; nay, in some of the separate years, their proportion rises even as high as a fifth; while, in the second decennium, the average proportion of cases of ague is only $\frac{1}{600}$th of the general mass of disease. The author accounts for this immense difference by improvements on the land, as drainage, cultivation, and by the superior construction of houses. In the first period, fever has a proportion of 12·71, and a mortality of 0·68; in the second, the proportion is 7·36, and the mortality 0·34. Smallpox is greatly less in the second period. Of the remaining epidemical diseases, in all, with the exception of erysipelas, the proportion is higher in the second than in the first period.

From a table showing the proportion per cent of diseases of the nervous apparatus to all others, we find that the amount of relative prevalence in the first decennium was nearly double that of the second. Yet, on further calculation, it appears that the relative mortality exceeded that of the first in the proportion of 20 to 15.

From a table showing the proportion per cent. of diseases of the respiratory apparatus to all others, we find that maladies of this sort constituted, at an average, during the first decennium, nearly a tenth of the whole cases brought under treatment; and that, in the second decennium, their prevalence was slightly increased. Of 4·60, the average mortality per cent. during the first period, 1·09 or nearly a fourth was owing to this class of ailments; while, in the second period, they produced fatal results in only 0·67 per cent.; thus still retaining, however, nearly the proportion of one fourth the average mortality per cent. from all diseases of the latter period.

From a table showing the proportion per cent. of disease of the circulatory apparatus to all other diseases, we find that the prevalence of the former has been five times greater in the second period than in the first, yet still amounting to a very insignificant proportion. The mortality in the second period is 0·09 per cent. of all diseases treated. In the first, no affection of this class has been assigned as a cause of death.

From a table showing the proportion per cent. of diseases of the digestive organs to all other diseases, we learn that this class of diseases has been nearly twice as prevalent in the second period as in the first; the mean proportion in the first having been 28·34; and in the second 15·77 per cent. upon all kinds of diseases taken collectively. The difference of mortality in the two decennia, from diseases of this class, is most remarkable. In the earlier one, of 100 cases of diseases of the digestive organs, 6·73 ended fatally; in the second only 1·44 proved mortal.

Regarding the seasons of the year, in which the greatest mortality prevailed, the record enables the author to offer little information in regard to the first period, and none whatever in regard to the second. Of 100 deaths recorded in the first six years of the first decennium, 26·72 occurred in spring, 30·18 in summer, 12·24 in autumn, and 25·86 in winter.—Dr. Charles Wilson, *Quarterly Journal of Agriculture*, No. lv. *Dec.* 1841.

